# Psychotherapeutic interventions for depressive symptoms in older adults in a community setting: a systematic review protocol

**DOI:** 10.3389/fpsyt.2024.1448771

**Published:** 2024-08-09

**Authors:** Bruno Morgado, Celso Silva, Inês Agostinho, Filipe Brás, Pedro Amaro, Leonel Lusquinhos, Brooke C. Schneider, Cesar Fonseca, Núria Albacar-Riobóo, Lara Pinho

**Affiliations:** ^1^ Department of Nursing, Universitat Rovira e Virgilli, Tarragona, Spain; ^2^ Polytechnic Institute of Portalegre, Portalegre, Portugal; ^3^ Comprehensive Health Research Centre, Évora, Portugal; ^4^ Beja School of Health - Polytechnic Institute of Beja, Beja, Portugal; ^5^ West Lisbon Local Health Unit, EPE., Lisbon, Portugal; ^6^ Alto Alentejo Local Health Unit, Portalegre, Portugal; ^7^ São João de Deus School of Nursing, Évora, Portugal; ^8^ University Medical Centre Hamburg-Eppendorf, Hamburg, Germany

**Keywords:** psychotherapies, non-pharmacological therapy, non-pharmacological treatment, depression, older adults, community setting, systematic review, protocol Português (Portugal)

## Abstract

Depression is a prevalent illness among the elderly, so psychotherapeutic interventions are needed to promote health and quality of life. This literature review aims to identify trials of any psychotherapeutic interventions aimed at reducing depressive symptoms in older adults. A systematic literature review with a meta-analysis will be carried out. The following databases will be searched: CINAHL Plus with Full Text, MedicLatina, MEDLINE with Full Text, and Psychology and Behavioral Sciences Collection. To minimize bias, four reviewers will independently assess the inclusion of studies. The fifth reviewer will be responsible for disaggregating inclusion and exclusion between two authors. The results of the analysis will be grouped in a table with the characteristics of the included studies, including author, year, sample, objectives, methods, assessment instruments, psychotherapeutic interventions, results, follow-up and a data compilation scheme. This scientific article is a systematic review protocol for which the data has already been extracted and is being analyzed. Examples of possible strategies to include cognitive restructuring and mental health programs for older adults in community settings. With this clear and direct identification of psychotherapeutic interventions, it will be clearer for mental health professionals to be able to intervene effectively to promote the mental health and well-being of older adults.

**PROSPERO registration number:**

CRD42023449190

## Introduction

The world is experiencing a remarkable demographic shift, characterized by a substantial increase in the aging population. This global phenomenon is primarily attributed to declining birth rates and increased life expectancy. While this aging trend represents a significant triumph for healthcare and living standards, it also brings unique challenges. One such challenge is the heightened incidence of depression among the older adults (1).

According to the World Health Organization (WHO), the global population of individuals aged 60 and above is expected to double by 2050. This demographic shift is not uniform; it varies across regions and countries. Nations like Japan, Italy, and Germany are witnessing particularly rapid aging trends. While this demographic transformation is an indicator of improved healthcare and living conditions, it also brings forth a range of health-related challenges, chief among them being mental health issues, notably depression (1, 2).

Depression is often considered a natural part of the aging process, but it is essential to recognize that it is not an inevitable consequence of growing older (3). The prevalence of depression among the older adults is alarming, with estimates suggesting that up to 15% of older adults experience depressive symptoms (3–5). It’s important to clarify that Depression is a diagnosable mental disorder, while depressive symptoms are isolated and occasional signs, such as depressed mood and anhedonia (6). These symptoms, if left unaddressed, can have severe consequences, including a reduced quality of life, increased morbidity, and even a higher risk of mortality (7). (4, 8–10). Depression treatment is also important because it is one of the main causes of years lived with disability (YLDs) (11, 12).

Depression in older adults is associated with great suffering, with impaired functioning in daily life, with low remission and suicide (13).

Older adults are particularly vulnerable to the determinants of depression, with depression being associated with cognitive deficits and an increased risk of dementia (14).

Factors Contributing to Depression in the older adults:

1. Social Isolation: Many older adults face social isolation due to the loss of friends and family members, retirement, or physical limitations. This isolation can lead to feelings of loneliness and depression.
2. Health Issues: Aging often comes with an increased burden of chronic illnesses, pain, and disabilities. Coping with these health challenges can trigger or exacerbate depression.
3. Loss and Grief: The older adults often face the loss of loved ones, which can be emotionally devastating. Grief and bereavement are powerful triggers for depression.
4. Neurobiological Changes: Aging is associated with neurobiological changes in the brain, which can make individuals more susceptible to mood disorders (2, 4, 15).

While medications can be effective in treating depression, they may not always be the best option, especially for older adults who may already be taking multiple medications for other health conditions (16). Psychotherapeutic interventions therapies offer a safer and more holistic approach to addressing depression in the older adults:

1. Psychotherapy: Various forms of psychotherapy, such as cognitive-behavioral therapy (CBT) and interpersonal therapy (IPT), have proven effective in treating depression in older adults. These therapies provide coping strategies, emotional support, and a safe space to discuss their concerns (17).
2. Physical Activity: Regular exercise has been shown to have a significant positive impact on mood. Encouraging older adults to engage in physical activities can help alleviate depressive symptoms (7).
3. Social Support: Addressing social isolation is crucial. Community programs, support groups, and fostering social connections can reduce feelings of loneliness and depression (18).
4. Mindfulness and Meditation: Mindfulness-based practices can enhance emotional well-being. Techniques like meditation and yoga can help manage depressive symptoms and improve overall mental health (19).
5. Art and Music Therapy: Creative outlets, such as art and music therapy, can provide a sense of purpose and joy, combating depression in the older adults (20).

The increasing global aging population is undeniably linked to a rising incidence of depression among older adults (1, 3, 15). Psychotherapeutic interventions therapies offer a ray of hope in addressing this concerning issue. By promoting social engagement, physical activity, psychotherapy, and creative outlets, we can enhance the mental well-being of our older adults population and ensure that they enjoy their golden years with dignity and happiness (18). It is essential for societies worldwide to recognize the importance of these psychotherapeutic interventions and integrate them into healthcare systems to provide comprehensive care for our aging population (16).

Although there is evidence supporting the efficacy of psychotherapeutic interventions in reducing depression among older adults, a thorough search in databases such as the Cochrane Database, CINAHL, PubMed, and PROSPERO reveals that many evaluated interventions predominantly target dependent older adults, most of whom are institutionalized. This highlights a significant research gap regarding psychotherapeutic interventions for older adults within a community context.

A preliminary search of the PUBMED database using the Boolean equation (“Depression AND Non pharmacologic interventions AND Older Adults AND Community AND systematic review”) yielded 25 results. None of these studies focused exclusively on psychotherapeutic interventions for older adults with depression in a community setting.

Our review distinctly differs from existing studies by concentrating specifically on non-pharmacologic, psychotherapeutic interventions for older adults experiencing depression within their communities. By focusing on this demographic, we aim to address the unique challenges and dynamics faced by older adults living independently, thereby filling a critical gap in current research. This approach not only underscores the necessity of our review but also emphasizes its originality, as it seeks to provide targeted insights and recommendations that are currently lacking in the literature.

Our review aims to bridge this gap by exclusively examining community-based psychotherapeutic interventions for older adults with depression, thus contributing valuable, previously unexplored knowledge to the field. This article reflects what is a recorded protocol of a systematic literature review with meta-analysis of the data, going through its essential stages. By writing this article, we aim to demonstrate the rigor, clarity and quality of the whole review process. As a result of this systematic review with meta-analysis, we believe we can contribute to knowledge about improving and maintaining the well-being and quality of life of older people and identify psychotherapeutic interventions that have an impact on the mood of older people, thus promoting rigorous practice based on scientific evidence.

### Objectives

This literature review aims to identify trials of any psychotherapeutic interventions aimed at reducing depressive symptoms in older adults. Examples of possible strategies to include cognitive restructuring and mental health programs for older adults in community settings.

### Review questions

What psychotherapeutic interventions contribute to the reduction of depressive symptoms in the older adults in a community environment?

## Methods and analysis

This is Systematic review protocol was developed in accordance with Preferred Reporting Items for Systematic Reviews and Meta-Analyses (PRISMA) (21) and was registered in PROSPERO under registration number CRD42023449190.

Considering that the scope of this study is very specific and still understudied, in the sense that we can easily identify isolated psychotherapeutic interventions with an effect on reducing depressive symptoms, but a review that compiles the most effective ones we have not found, just as there is no previous record in the PROSPERO database, our review being the first to be registered. We chose to include only Randomized Controlled Trials (RCTs), because RCTs are considered higher evidence studies because they eliminate bias, randomizing participants and controlling variables, ensuring more reliable and accurate results.

## Eligibility criteria

### Population

The inclusion criteria are older adult patients aged 60 or over with depressive symptoms. The exclusion criteria are people under the age of 60 and studies that are not RCTs.

### Intervention

This literature review will exclusively consider randomized controlled trials (RCTs) evaluating the effectiveness of any psychotherapeutic interventions that reduce depressive symptoms in older adults.

The justification for including only Randomized Controlled Trials (RCTs) in this literature review stems from the rigorous methodological standards that RCTs uphold, which are considered the gold standard for evaluating the effectiveness of interventions. RCTs offer the highest level of evidence due to their ability to minimize bias through randomization and the use of control groups. This methodological strength allows for a clearer determination of causality between the intervention and the observed outcomes, thereby providing more reliable and valid results.

Excluding other study designs, such as observational studies, case-control studies, and cohort studies, strengthens the validity of our review’s findings. While these designs can offer valuable insights, they are more susceptible to various biases, such as selection bias, recall bias, and confounding variables, which can compromise the accuracy of the results. By focusing exclusively on RCTs, our review aims to ensure that the conclusions drawn are based on the most robust and credible evidence available.

The specificity of our study’s scope is defined by its aim to identify trials on any psychotherapeutic interventions that effectively reduce depressive symptoms in older adults. This focus is crucial because older adults often face unique psychological, social, and physical challenges that can influence the manifestation and treatment of depression. By narrowing our scope to psychotherapeutic interventions, we intend to comprehensively evaluate the effectiveness of various therapeutic approaches, such as cognitive-behavioral therapy, interpersonal therapy, and mindfulness-based interventions, specifically within this demographic.

Furthermore, our study’s emphasis on community-dwelling older adults highlights an often-overlooked population in existing research. While many studies target institutionalized older adults or those with significant dependencies, our review seeks to address the gap in understanding the impact of psychotherapeutic interventions on those living independently in the community. This distinction is vital, as community-dwelling older adults may have different support systems, levels of autonomy, and social interactions, all of which can influence the effectiveness and feasibility of psychotherapeutic interventions.

### Comparison

This review will only include studies with a control group (Treatment as usual) and an experimental group, subject to sample randomization.

### Primary outcome

This literature review will consider randomized controlled trials of any psychotherapeutic interventions that decrease depressive symptomatology in older adults. Psychotherapeutic interventions can include cognitive-behavioral therapy (CBT), interpersonal therapy (IPT), and mindfulness-based stress reduction (MBSR). Clinical assessment tools for evaluating depression in older adults can include the Beck Depression Inventory (BDI), Geriatric Depression Scale (GDS), and the Hamilton Depression Rating Scale (HDRS).

### Secondary outcome

Considering the extracted data and understanding how depression levels will be assessed, and which specific depressive symptoms will be reduced, we construct secondary outcomes, considering which clinical assessment instruments are used to measure depression levels and what are the main results after application. of psychotherapeutic intervention, ruminative thoughts were reduced, for example.

### Study design

This review will only include studies with a control group and an experimental group, with the sample randomized by group. With the synthesis of the data, statistical tests will be applied, explained later in this article, in order to fulfill the meta-analysis of the data, increasing the evidence of our conclusions.

### Context

All studies related to trials of any psychotherapeutic interventions that decrease depressive symptomatology on older adults aged 60 years and over with any kind of depression, only in the community context, will be included in this review.

## Search strategy

### Data sources

The search engine used will be EBSCO and the databases selected will be CINAHL Plus with full text, MedicLatina, MEDLINE with full text, Psychology and Behavioral Sciences Collection, PUBMED and Scopus.

### Search terms

To search for results, the Boolean equation will include the concepts combined according to the Medical Subject Headings (MeSH) terms:

(“Depression”) OR (“Depressive Disorder”) OR (“depressive symptoms”) OR (“major depressive disorder”) AND (“Psychotherapy”) OR (“cognitive behavioral therapy”) OR (“psychotherapy groups”) OR (“psychotherapy, brief”) OR (“behavior therapy”) OR (“non-pharmaceutical”) OR (“non-pharmaceutical interventions”) OR (“non-pharmaceutical treatment”) AND (“community”) OR (“home care”) OR (“home care services”) OR (“home health care”) OR (“home healthcare”) OR (“home nursing”) AND (older adult or elderly or geriatric patients).

## Data collection and analysis

### Selection of studies and data extraction

This literature review will consider randomized controlled trials of any psychotherapeutic interventions that decrease depressive symptomatology in older adults. All RCTs with interventions that reduce depressive symptoms in older adults, i.e. over 60 years of age in a community setting, will be selected for inclusion.

The studies resulting from the search in each database will be exported to Mendeley, that is a reference management tool and academic social network that helps researchers organize their research, collaborate online, and discover the latest findings. It allows users to manage and share research papers, discover research data, and collaborate online. With Mendeley, users can automatically generate bibliographies, import papers from other research software, and find relevant articles based on their reading habits. Duplicates will be removed, then given to the different authors to analyze and select. One author will have all the results, and three authors will split them up.

The results of the analyses will then be cross-referenced, and in the event of disagreement about the inclusion of a particular article, a fifth author will carry out the tie-breaker analysis, depending on the quality of the article and the inclusion criteria.

The authors will identify the articles that should be included by reading the Titles, Abstracts and full manuscripts, selecting those that are RCTs, the population is over 60 years of age, compare the effectiveness of a psychotherapeutic intervention, in the experimental group, with the usual treatment, of the control group. Extracting into the summary tables, the psychotherapeutic intervention and the clinical assessment instruments used to measure depression levels.

### Quality appraisal

As this is a review of RCTs, the quality assessment tool for each selected article will be carried out using the Joanna Briggs Institute assessment tools to help assess the reliability, relevance, and results of the published articles (22).

To minimize bias, four reviewers will independently assess the inclusion of studies by reading the title, abstracts and keywords and exclude those that do not meet the inclusion criteria for this review. The fifth reviewer should be consulted in case of disagreement or doubt. The fifth reviewer will be consulted in the event of a disagreement or doubt, where a tiebreaker will be made as to whether or not the article should be included, based on the quality of the article and the inclusion criteria. Subsequently, the full texts will be assessed by the ten researchers. In order to present this selection process, the PRISMA flowchart will be presented with the results of the screening in the different phases.

### Strategy for data synthesis

A synthesis will be carried out with Meta-Analysis. The data that will be taken from the selected articles will address the psychotherapeutic interventions that were implemented to reduce depressive symptoms in older adults, the clinical assessment instruments used to evaluate depressive symptoms, the results of the implemented interventions, that is, whether there are evidence that the interventions reduced depressive symptoms or not, and the nature of the intervention implemented, that is, whether it is a group or individual intervention.

A data synthesis table will be constructed to summarize the answers to the research question.

A summary table of the selected articles will also be created with information regarding the author, the year, the sample, the objectives of the study, the method, the evaluation instruments, the psychotherapeutic interventions, the results, and the monitoring.

Subsequently, a narrative synthesis of the results will be conducted to address the scope and objectives of this systematic review.

For each comparison and outcome, a description of the synthesized results and the certainty of the results will be provided, and which studies contribute to the synthesis will be indicated.

The data extracted from the RCTs was analyzed using IBM^®^ SPSS^®^ Statistics V28 software. The random effects model will be used, based on the assumption of heterogeneity of the studies collected, generating more conservative estimates of precision, with weighting based on the inverse variance method in which studies with lower variance (more precise) are given greater weight (23). To assess the effect of psychotherapeutic interventions on the study population, the standardized difference in means will be used as a measure of effect (Hedges adjusted g), with standard error adjustment. This adjustment allows greater weight to be given to studies with lower standard error in the meta-analysis (24).

Hedges’ g provides a more conservative estimate, useful in small samples, with an effect interpretation: 0.2 - small; 0.5 - medium; 0.8 - large (23). To quantify the degree of variability in the whole sample and in the subgroups, homogeneity estimates will be calculated. A statistically significant value (p<0.05) will suggest that there is heterogeneity. I2 estimates will represent the proportion of variability between studies that is not due to chance (random). The magnitude of I2 will be interpreted as: 0% to 40% may not be important; 30% to 60% may represent moderate heterogeneity; 50% to 90% may represent substantial heterogeneity; 75 to 100% will represent considerable heterogeneity (23). The significance of the observed I2 value will assess by using the Q statistic (Chi-Square), where a p-value of <0.05 will suggests homogeneity. Publication bias will be assessed using the Egger test. In the measurement instruments used in the studies, higher scores (mean) suggest greater depressive symptomatology.

Subgroup analysis (group vs individual Psychotherapeutic interventions) will be carried out to investigate the effects on the primary outcome. Subgroup analysis will allows for a more robust analysis of the relationship between the intervention and the results obtained (23).

### Assessment of the quality of the evidence produced by the review.

We will trace the psychotherapeutic interventions that have been implemented to reduce depressive symptoms in the older adults regardless of their effectiveness, minimizing publication bias, the clinical assessment instruments used to assess depressive symptoms, the results of the implemented interventions and the nature of each psychotherapeutic intervention carried out.

To minimize bias, four reviewers will independently assess the inclusion of studies by reading the title, abstracts and keywords and will exclude those that do not meet the inclusion criteria for this review. The fifth reviewer must be consulted in case of disagreement to break the tie based on the quality of the article and the inclusion criteria. Subsequently, the complete texts will be evaluated by the ten researchers.
